# From foreign language classroom anxiety to English learning engagement: the roles of cognitive reappraisal and expressive suppression

**DOI:** 10.3389/fpsyg.2026.1754113

**Published:** 2026-03-10

**Authors:** Chen Chen, Zikai Guo

**Affiliations:** School of Foreign Studies, Xi'an Medical University, Xi'an, China

**Keywords:** cognitive reappraisal, emotion regulation, English learning engagement, expressive suppression, foreign language classroom anxiety

## Abstract

**Background:**

Emotions play a central role in second and foreign language (L2) learning, yet relatively little is known about how specific emotion regulation strategies shape the link between foreign language classroom anxiety (FLCA) and learners' engagement. Drawing on the process model of emotion regulation and positive psychology in second language acquisition (SLA), this study examined whether cognitive reappraisal and expressive suppression in English learning mediate the association between FLCA and English learning engagement among Chinese university students.

**Methods:**

A cross-sectional online questionnaire survey was administered to 260 undergraduates enrolled in compulsory College English courses at multiple universities in Mainland China. Students completed newly developed, context-specific scales assessing FLCA, cognitive reappraisal in English learning, expressive suppression in English learning, and English learning engagement, along with demographic and English-learning background items. Descriptive statistics and correlations were computed, a four-factor confirmatory factor analysis evaluated the measurement model, and a parallel mediation model tested the hypothesized paths.

**Results:**

Students reported moderate levels of FLCA and expressive suppression, moderate use of cognitive reappraisal, and moderately high English learning engagement. FLCA correlated negatively with both cognitive reappraisal and engagement, and positively with expressive suppression. In the structural model, FLCA showed a substantial negative total effect on engagement. When mediators were included, higher FLCA predicted less frequent cognitive reappraisal and more frequent expressive suppression; in turn, cognitive reappraisal was positively, and expressive suppression negatively, associated with engagement. Both indirect paths were statistically significant, and together accounted for a considerable portion of the FLCA–engagement link, while a meaningful direct effect of FLCA on engagement remained.

**Conclusion:**

The findings indicate that foreign language classroom anxiety is robustly and negatively related to English learning engagement and that this relationship is partly explained by students' emotion regulation strategies. Cognitive reappraisal in English learning functions as an adaptive pathway that helps sustain engagement, whereas expressive suppression operates as a maladaptive pathway associated with disengagement. These results highlight the value of classroom practices and institutional initiatives that not only reduce excessive anxiety but also explicitly foster adaptive emotion regulation in university English-as-a-foreign-language contexts.

## 1 Introduction and literature review

### 1.1 Introduction

In recent decades, research in second and foreign language (L2) education has increasingly recognized that learners' emotions play a central role in shaping their experiences and outcomes. Among negative emotions, foreign language classroom anxiety (FLCA) has been identified as a distinct and pervasive construct that can interfere with language processing and performance (Horwitz et al., 1986). Meta-analytic and review evidence indicates that higher FLCA is consistently associated with lower achievement and less favorable self-perceptions in the L2 (Botes et al., 2020; Shen, 2021). In parallel, the positive psychology movement has highlighted learners' engagement, a positive, fulfilling state characterized by vigor, dedication, and absorption, as a key driver of academic success (Schaufeli et al., 2002; Zeng, 2021). Understanding how anxiety and engagement are linked, and which psychological processes may bridge them, is therefore an important task for both theory and practice in L2 education.

### 1.2 Literature review

#### 1.2.1 Foreign language classroom anxiety and learning engagement

FLCA refers to the tension and apprehension specifically associated with foreign language learning in classroom settings (Horwitz et al., 1986). Anxious learners often report worry about negative evaluation, fear of making mistakes, and discomfort when speaking in front of others. Building on the Foreign Language Classroom Anxiety Scale (FLCAS), recent work has refined measurement and confirmed that even short forms capture a powerful negative emotional state that can undermine learning (Botes et al., 2022). FLCA has been linked to lower self-perceived proficiency, reduced willingness to communicate, and poorer performance in speaking, listening, and overall achievement (Botes et al., 2020; Tsang and Dewaele, 2024). Recent evidence among Chinese undergraduate EFL learners further suggests that foreign language anxiety is closely implicated in learners' willingness to communicate and related motivational–affective processes (Li, 2025).

Learning engagement, in contrast, is a positive motivational–affective state characterized by energy, involvement, and persistence in learning tasks. The Utrecht framework conceptualizes engagement as comprising vigor, dedication, and absorption (Schaufeli et al., 2002). In school and university contexts, higher engagement predicts better grades and achievement, partly because engaged students invest more sustained effort and show greater persistence (Carmona-Halty et al., 2019). In L2 learning, reviews suggest that positive emotions such as enjoyment and engagement tend to co-occur and are associated with more frequent and risk-taking use of the target language, whereas anxiety is generally negatively related to engagement and attainment (Shen, 2021; Zeng, 2021; Tsang and Dewaele, 2024). These findings point to a robust negative association between FLCA and engagement, but they do not yet fully explain the psychological mechanisms that connect them. Although existing evidence supports a robust negative association between FLCA and engagement, this relationship is often treated as a descriptive regularity rather than being explained in process terms. In particular, engagement reflects sustained energy and involvement in English learning, yet the literature has offered less specificity regarding the proximal psychological processes through which classroom anxiety may translate into learners' day-to-day vigor, dedication, and absorption. Addressing this gap requires attention to how learners manage anxiety as it unfolds in learning situations, which provides a clearer basis for understanding why some anxious learners disengage while others maintain involvement. This consideration motivates our focus on emotion regulation strategies as a plausible mechanism linking FLCA to English learning engagement.

At the same time, the directionality between FLCA and language achievement is not settled. Beyond evidence positioning anxiety as a factor that can hinder performance, longitudinal findings also suggest that achievement-related experiences may shape subsequent anxiety. For instance, cross-lagged panel evidence indicates that earlier language achievement can predict later anxiety more consistently than anxiety predicts later achievement (Alamer and Lee, 2024). Related longitudinal work likewise shows that first-language achievement can precede changes in second-language reading anxiety (Sparks and Alamer, 2023). Taken together, these results point to the possibility of reciprocal or feedback processes linking performance and anxiety. In the present study, we focus on FLCA as a proximal classroom-specific emotional experience that may be associated with English learning engagement through emotion regulation strategies; however, we avoid causal language and recognize that longitudinal designs are required to evaluate alternative temporal pathways.

#### 1.2.2 Emotion regulation in academic and language learning contexts

Emotion regulation refers to the processes through which individuals influence which emotions they have, when they have them, and how they experience and express these emotions. Within the process model, two widely studied strategies are *cognitive reappraisal* and *expressive suppression* (Gross and John, 2003). Cognitive reappraisal involves changing how one interprets a situation to alter its emotional impact (e.g., viewing a demanding language task as a challenge and an opportunity to learn), whereas expressive suppression involves inhibiting the outward signs of emotion after it has been generated (e.g., hiding nervousness while remaining highly anxious internally). A large body of research suggests that these strategies are not equally adaptive. Meta-analytic and review work shows that habitual use of cognitive reappraisal is generally associated with greater wellbeing, more positive affect, and better interpersonal and academic outcomes, whereas habitual use of expressive suppression tends to relate to more negative affect, lower wellbeing, and impaired social functioning (Aldao et al., 2010; Webb et al., 2012; Cutuli, 2014). In educational settings, empirical evidence suggests that students who report greater use of cognitive reappraisal tend to show more adaptive coping and higher engagement-related outcomes (Buzdar and Ikram, 2024). By contrast, expressive suppression has been shown to carry cognitive costs (e.g., poorer memory for learning materials under suppression instructions) (Dunn et al., 2009), which may constrain learners' effective participation and sustained involvement when emotional demands are high.

Emerging evidence in language education mirrors these patterns. Studies have shown that positive emotions such as foreign language enjoyment are linked to learners' engagement and achievement, whereas anxiety and other negative emotions undermine engagement (Zeng, 2021; Tsang and Dewaele, 2024). However, explicit work on how L2 learners regulate their emotions, and how particular strategies such as cognitive reappraisal and expressive suppression relate to classroom anxiety and engagement, remains relatively scarce. At the same time, broader higher-education research indicates that cognitive reappraisal can mediate the impact of stressors on student engagement in online and face-to-face learning contexts (e.g., Buzdar and Ikram, 2024), suggesting that emotion regulation may be a key mechanism linking negative experiences to academic involvement.

#### 1.2.3 Linking FLCA, emotion regulation, and learning engagement

The links among foreign language classroom anxiety (FLCA), emotion regulation, and engagement can be understood as a set of mutually reinforcing affective and behavioral processes in learning contexts. Recent L2 research indicates that higher anxiety is commonly accompanied by avoidance tendencies such as reduced willingness to communicate, lower participation, and less sustained involvement in language learning activities, all of which are conceptually close to the behavioral and energetic facets of engagement (Tsang and Dewaele, 2024; Zhao, 2023). From an emotion-regulation perspective, learners facing anxiety-provoking classroom demands may differ in how they manage their emotional responses, and these differences can shape whether they remain vigorous and involved or gradually disengage. In higher-education and language-learning–adjacent evidence, cognitive reappraisal has been linked to more adaptive learning-related functioning and greater engagement, whereas a heavier reliance on suppressing emotional expression tends to relate to poorer adjustment and lower engagement-related outcomes (Buzdar and Ikram, 2024; Huang et al., 2023; Iuga et al., 2025). Bringing these strands together, we propose that FLCA may undermine engagement partly by making constructive re-interpretation of stressful learning situations less likely, while simultaneously increasing reliance on suppression as a response-focused strategy. This integrative framing provides the rationale for testing a parallel mediation model in which cognitive reappraisal and expressive suppression jointly account for the association between FLCA and English learning engagement.

From this perspective, cognitive reappraisal can be conceptualized as a potentially adaptive pathway through which students manage FLCA and remain engaged, whereas expressive suppression may constitute a maladaptive pathway that exacerbates the negative impact of FLCA on engagement. Clarifying the relative contributions of these two strategies is particularly relevant in university English-as-a-foreign-language (EFL) settings, where students face high-stakes exams, demanding curricula, and increased autonomy in managing their emotions and learning behaviors.

### 1.3 Theoretical and pedagogical contributions of the present study

Informed by the above literature, the present study examines Chinese university students learning English as a foreign language and tests a parallel mediation model linking foreign language classroom anxiety (FLCA) to English learning engagement through two emotion regulation strategies enacted in English learning: cognitive reappraisal and expressive suppression. Specifically, we investigate whether higher FLCA is associated with lower engagement and whether this association can be partly explained by reduced use of cognitive reappraisal and increased use of expressive suppression. By focusing on engagement as a sustained positive learning state and by conceptualizing reappraisal and suppression as strategies deployed in English-learning situations, this study offers a process-oriented account of how classroom anxiety may translate into learners' day-to-day vigor, dedication, and absorption, with implications for pedagogical support aimed at anxious learners' sustained involvement in English learning. Based on this framework, we formulate the hypotheses below and test them using structural equation modeling.

### 1.4 Hypotheses

The present cross-sectional study focuses on Chinese university students learning English as a foreign language. We conceptualize FLCA as a negative emotion specific to the foreign language classroom, cognitive reappraisal as an adaptive emotion regulation strategy applied to English learning, expressive suppression as a response-focused strategy of inhibiting emotional expression in English learning, and learning engagement as a positive, fulfilling state characterized by vigor, dedication, and absorption in English learning.

**Figure 1:**
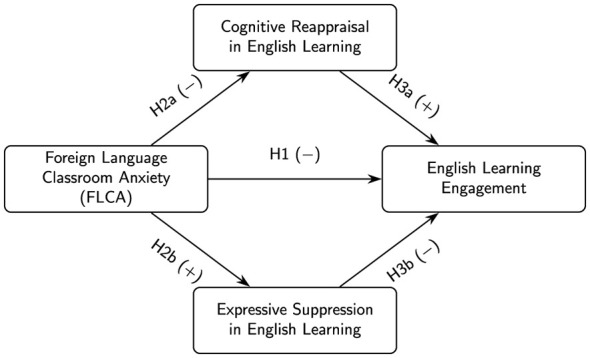
Conceptual model of the hypothesized relationships among foreign language classroom anxiety (FLCA), cognitive reappraisal, expressive suppression, and English learning engagement.

Drawing on the literature reviewed above, we propose a parallel mediation model in which cognitive reappraisal and expressive suppression jointly link FLCA to engagement (Figure 1). In this model, FLCA is placed on the left, English learning engagement on the right, and the two emotion regulation strategies form an upper and a lower pathway between them. The central horizontal arrow (H1) represents the expected direct negative association between FLCA and engagement: higher anxiety is hypothesized to be associated with lower engagement in English learning. The upper pathway captures the role of cognitive reappraisal. We hypothesize that higher FLCA is associated with less frequent use of cognitive reappraisal in English learning (H2a), and that greater use of cognitive reappraisal is in turn associated with higher engagement (H3a). Together, these two arrows imply an indirect pathway (H4a) whereby FLCA undermines engagement partly by making it more difficult for students to reinterpret emotionally challenging situations in a constructive way. The lower pathway captures the role of expressive suppression. We expect that higher FLCA is associated with greater use of expressive suppression in English learning (H2b), and that more frequent suppression is associated with lower engagement (H3b). This yields a second indirect pathway (H4b) in which FLCA reduces engagement by promoting a less adaptive, response-focused strategy of hiding emotional reactions in class. Overall, the model assumes that FLCA will be negatively related to engagement both directly (H1) and indirectly via these two distinct emotion regulation routes (H4a and H4b).

We tested this parallel mediation model using structural equation modeling (SEM) with latent variables representing FLCA, cognitive reappraisal, expressive suppression, and English learning engagement.

## 2 Methods

### 2.1 Participants

Participants were 260 undergraduate students recruited from four universities in Xi'an, Shaanxi Province, China: Xi'an Jiaotong University, Northwest University, Shaanxi Normal University, and Xi'an Medical University. By including institutions that range from elite research-oriented universities to specialized medical and teacher-training colleges, the sample encompasses a broad spectrum of undergraduate EFL learners across various academic disciplines, including medicine, engineering, humanities, and social sciences. At these institutions, Mandarin Chinese is the primary medium of instruction for major academic subjects. For College English courses, a mixed instructional mode is typically employed, where Mandarin is used for explanation and management, while English is used for language input and practice activities in accordance with the National College English Teaching Guidelines. All questionnaires were completed individually via an online survey platform during regular class time. An instructed-response item was included as an attention check. Cases with substantial missing data, failure on the attention-check item, or clear patterned responding were excluded from the analyses. After data screening, a total of 260 students remained in the final sample. On average, participants were late adolescents or young adults and had several years of prior English learning experience in formal schooling.

Descriptive information on participants' demographic and English-learning-related characteristics is presented in Table 1. The final sample had a mean age of 20.9 years (*SD* = 1.16). Approximately two thirds of the sample were female (66.9%). Students were distributed across Years 1–4, with 61.5% in Years 1–2. With respect to academic background, 43.1% were enrolled in language-related majors and 56.9% in non-language majors. Self-rated English proficiency clustered around the middle of the scale (“fair” = 43.1%; “good” = 26.9%). Most participants reported substantial prior exposure to English, with over 80% reporting at least 6 years of formal English learning and 46.2% reporting 10 years or more. The majority had taken at least one major English examination (most commonly CET-4 or CET-6). Weekly English learning time was heterogeneous across formal instruction and extracurricular learning, and 9.2% reported overseas study/living experience in an English-speaking environment.

**Table 1:** Demographic and learning-related characteristics of the sample (*N* = 260).

**Variable**	**Category**	***n* (%) or *M* (*SD*)**
Age (years)	*M* (*SD*)	20.9 (1.16)
Gender	Female	174 (66.9%)
	Male	86 (33.1%)
Year of study	Year 1	72 (27.7%)
	Year 2	88 (33.8%)
	Year 3	62 (23.8%)
	Year 4	38 (14.6%)
Major type	Language-related	112 (43.1%)
	Non-language	148 (56.9%)
Self-rated English proficiency	Very poor	12 (4.6%)
	Poor	46 (17.7%)
	Fair	112 (43.1%)
	Good	70 (26.9%)
	Very good	20 (7.7%)
Overseas study/living experience	Yes	24 (9.2%)
	No	236 (90.8%)
Years of learning English	< 3 years	4 (1.5%)
	3–5 years	40 (15.4%)
	6–9 years	96 (36.9%)
	≥10 years	120 (46.2%)
Most recent major English exam	CET-4	124 (47.7%)
	CET-6	54 (20.8%)
	TEM-4	18 (6.9%)
	TEM-8	4 (1.5%)
	IELTS	10 (3.8%)
	TOEFL	6 (2.3%)
	Other	8 (3.1%)
	None	36 (13.8%)
Weekly hours of formal English classes	0–2 h	66 (25.4%)
	3–5 h	132 (50.8%)
	6–8 h	44 (16.9%)
	≥9 h	18 (6.9%)
Weekly hours of extracurricular English learning	Almost none	50 (19.2%)
	< 2 h	84 (32.3%)
	2–4 h	72 (27.7%)
	5–7 h	38 (14.6%)
	≥8 h	16 (6.2%)

Values are frequencies and percentages for categorical variables and mean (standard deviation) for age. Percentages may not total 100.0% due to rounding.

### 2.2 Measures

All questionnaires were administered in Chinese. The four focal scales (FLCA, cognitive reappraisal in English learning, expressive suppression in English learning, and English learning engagement) were developed and written directly in Chinese for the present study; therefore, no English-to-Chinese translation procedure was conducted. Prior to data collection, the Chinese item wording was reviewed and refined through expert review to improve clarity, readability, and content appropriateness for Chinese university EFL learners. Unless otherwise noted, all focal scale items were rated on a 5-point Likert scale ranging from 1 (strongly disagree) to 5 (strongly agree). The full item list, including the original Chinese items and their English renderings, is provided in Appendix 1.

#### 2.2.1 Foreign language classroom anxiety (FLCA)

FLCA was assessed with an 8-item self-report scale specifically developed for this study to capture university students' foreign language classroom anxiety in English classes. In constructing the items, we drew on the conceptualization of FLCA proposed by (Horwitz et al. 1986) and on subsequent work on short-form measures of FLCA (e.g., Botes et al., 2022), while adapting the wording to contemporary Chinese university EFL classrooms. The items tap fear of negative evaluation, communication apprehension, and test-related worry in English classes (e.g., “When the teacher calls on me in English class, I feel nervous”). Higher scores reflect higher levels of FLCA.

#### 2.2.2 Cognitive reappraisal in English learning

Cognitive reappraisal in English learning was measured with six items developed for this study to reflect students' tendency to reinterpret emotionally challenging English learning situations in a more adaptive way. Item content and phrasing were guided by the process model of emotion regulation and by the cognitive reappraisal construct as operationalized in the Emotion Regulation Questionnaire (ERQ; Gross and John, 2003). We did not translate the ERQ items verbatim; instead, we generated context-specific items in Chinese that operationalized the reappraisal construct in English-learning situations. A sample item is “When English learning makes me feel bad, I try to look at it from a different perspective.” Higher scores indicate more frequent use of cognitive reappraisal strategies in English learning.

#### 2.2.3 Expressive suppression in English learning

Expressive suppression in English learning was assessed with 6 items that were likewise developed for the present study. These items capture the extent to which students inhibit or hide their outward emotional expressions in English learning situations. The item pool was informed by the expressive suppression construct in the ERQ framework (Gross and John, 2003) and by broader work on response-focused emotion regulation, but the final wording was tailored to the EFL classroom context. Although the original ERQ suppression subscale contains four items, we used six items to enhance content coverage of suppression behaviors that commonly occur in English-learning contexts (e.g., deliberately concealing nervousness during classroom activities). Example items include “When I feel nervous in English class, I try not to show it to others.” Higher scores indicate more frequent use of expressive suppression strategies in English learning.

#### 2.2.4 English learning engagement

English learning engagement was measured with nine items developed to capture vigor, dedication, and absorption in English learning. The item development was informed by prior research on work and academic engagement, particularly the Utrecht Work Engagement Scale and its student version (UWES-S; Schaufeli et al., 2002; Carmona-Halty et al., 2019). We did not translate the full UWES-S item set; instead, we generated a context-specific Chinese item set designed to represent the three engagement facets in English-learning situations, using a concise 9-item format (three items per facet) to reduce respondent burden in the online survey. A sample item is “When I study English, I feel full of energy.” Higher scores indicate higher levels of engagement in English learning.

#### 2.2.5 Background variables

Part A of the questionnaire collected demographic and English-learning-related information. Specifically, students reported their gender (female, male, other/prefer not to say), age (in years), year of study (Year 1–4), and major type (language-related vs. non-language). They also indicated their self-rated English proficiency on a 5-point scale (1 = very poor, 5 = very good), whether they had any overseas study or living experience in an English-speaking country (yes/no), and the total number of years they had been learning English.

In addition, students reported their *most recent* major English examination taken [e.g., College English Test Band 4 (CET-4), College English Test Band 6 (CET-6), Test for English Majors Band 4 (TEM-4), Test for English Majors Band 8 (TEM-8), IELTS, TOEFL, other exam, or “none”]. They also indicated their average weekly hours of formal English instruction (e.g., compulsory English classes) and their average weekly hours of extracurricular English learning (e.g., self-study, online courses, English clubs). Finally, one instructed-response attention-check item [“To show that you are paying attention, please select ‘5 (strongly agree)' for this item.”] was placed immediately after the Cognitive Reappraisal scale (after CR6) and before the Expressive Suppression scale (before ES1). The full administration sequence was as follows: background questions, the FLCA scale, cognitive reappraisal, the attention check, expressive suppression, and English learning engagement.

### 2.3 Procedure

Data were collected via an online survey platform (Wenjuanxing) during regular class sessions. Students were told that participation was voluntary, responses would be anonymous, and they could withdraw at any time without penalty. The electronic informed consent was obtained: before accessing the questionnaire, participants were required to read a consent statement on the first page and click an “I agree to participate” option to signify their acknowledgment and voluntary participation. After providing informed consent, participants completed the background questions, followed by the FLCA, cognitive reappraisal, expressive suppression, and English learning engagement scales. The entire survey took approximately 10–15 min to complete. No course credits or monetary incentives were given, or minimal compensation was provided.

### 2.4 Data analysis

Data were analyzed using R (version 4.3.3, R Foundation for Statistical Computing, Vienna, Austria), including the lavaan package for structural equation modeling. All statistical tests were two-tailed with a significance level of α = 0.05.

**Figure 2:**
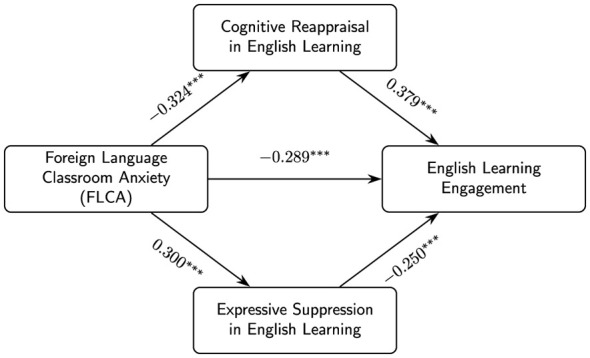
Structural model with standardized path coefficients. ^*^p < 0.05, ^**^p < 0.01, ^***^p < 0.001.

1. Data screening: we first screened the data and excluded cases with excessive missing responses, failure on the attention-check item, or clear patterned responding (e.g., identical answers across all items). For the remaining participants, missing values on individual items were handled using full information maximum likelihood (FIML) within the structural equation modeling framework.
2. Descriptive statistics: we then computed descriptive statistics for all study variables. For demographic and English-learning-related variables (e.g., gender, year of study, major type, years of learning English, recent English examinations, weekly hours of English learning), we calculated frequencies and percentages; these sample characteristics are summarized in Table 1. For the four focal psychological constructs, FLCA, cognitive reappraisal in English learning, expressive suppression in English learning, and English learning engagement, we computed means, standard deviations, and Pearson correlations among the composite scores; these results are presented in Table 2. These composite-score correlations are reported for descriptive purposes only and are not the basis of the SEM hypothesis tests.
3. Measurement and structural models: finally, we used structural equation modeling to examine the measurement and structural relations among the four latent constructs. All hypothesis-testing models were estimated as latent-variable SEMs: FLCA, cognitive reappraisal, expressive suppression, and engagement were modeled as latent factors indicated by their respective items (rather than as observed composite variables). As a first step, we specified a four-factor confirmatory factor analysis (CFA) with FLCA, cognitive reappraisal in English learning, expressive suppression in English learning, and English learning engagement as correlated latent variables. From this CFA, we obtained standardized factor loadings and latent-level reliability indices (composite reliability and average variance extracted) for each construct; these results are summarized in Table 3.
  On the basis of this measurement model, we then estimated two *nested* structural SEMs. Model A estimated the FLCA → engagement total-effect association *without specifying mediation paths*; that is, the mediator regressions (FLCA → cognitive reappraisal, FLCA → expressive suppression, cognitive reappraisal → engagement, and expressive suppression → engagement) were not estimated (fixed to zero). Model B was the parallel mediation model that added these four structural paths while retaining a direct path from FLCA to engagement to test for partial mediation. Importantly, Model A and Model B shared the same four-factor measurement model (item-to-factor specification) as the CFA and differed only in their structural regressions among the latent factors. Direct, indirect, and total effects were estimated using bias-corrected bootstrap confidence intervals based on 5,000 resamples. The standardized path coefficients and confidence intervals from these models are presented in Table 4, and the final structural model is illustrated in Figure 2.

**Table 2:** Descriptive statistics and correlations among key variables.

**Variable**	***M***	***SD***	**1**	**2**	**3**	**4**
1. FLCA	3.054	0.627	–			
2. Cognitive reappraisal	3.452	0.582	–0.324^***^	–		
3. Expressive suppression	3.108	0.639	0.300^***^	–0.097	–	
4. Learning engagement	3.598	0.611	–0.487^***^	0.497^***^	–0.374^***^	–

All variables are composite scale scores on a 1–5 Likert scale, with higher scores indicating higher levels of the construct. Values below the diagonal are Pearson correlations (*r*).

FLCA, foreign language classroom anxiety.

^*^p < 0.05, ^**^p < 0.01, ^***^p < 0.001.

**Table 3:** Measurement model: standardized factor loadings and internal consistency and convergent validity indices.

**Latent factor/item**	**Loading**	***SE***	***p***
**Foreign language classroom anxiety (FLCA)**			
FLCA1	0.712	0.049	<0.001
FLCA2	0.781	0.041	<0.001
FLCA3	0.738	0.043	<0.001
FLCA4	0.691	0.052	<0.001
FLCA5	0.814	0.039	<0.001
FLCA6	0.763	0.042	<0.001
FLCA7	0.684	0.051	<0.001
FLCA8	0.725	0.048	<0.001
**Cognitive reappraisal in English learning**			
CR1	0.703	0.050	<0.001
CR2	0.771	0.042	<0.001
CR3	0.729	0.044	<0.001
CR4	0.754	0.041	<0.001
CR5	0.802	0.038	<0.001
CR6	0.777	0.040	<0.001
**Expressive suppression in English learning**			
ES1	0.704	0.051	<0.001
ES2	0.768	0.044	<0.001
ES3	0.745	0.046	<0.001
ES4	0.789	0.041	<0.001
ES5	0.732	0.047	<0.001
ES6	0.758	0.043	<0.001
**English learning engagement**			
ENG1	0.724	0.043	<0.001
ENG2	0.792	0.038	<0.001
ENG3	0.832	0.032	<0.001
ENG4	0.776	0.039	<0.001
ENG5	0.815	0.034	<0.001
ENG6	0.761	0.041	<0.001
ENG7	0.743	0.040	<0.001
ENG8	0.691	0.052	<0.001
ENG9	0.707	0.049	<0.001
**Reliability indices (latent-level)**			
FLCA (CR, AVE)	CR = 0.892, AVE = 0.536		
Cognitive reappraisal (CR, AVE)	CR = 0.881, AVE = 0.552		
Expressive suppression (CR, AVE)	CR = 0.883, AVE = 0.557		
Engagement (CR, AVE)	CR = 0.934, AVE = 0.607		

Standardized loadings are from the four-factor confirmatory factor analysis.

FLCA, foreign language classroom anxiety; CR, composite reliability; AVE, average variance extracted.

**Table 4:** Structural model: direct, indirect, and total effects.

**Path/effect**	**Standardized**	***SE***	**95% CI**
**Model A: FLCA predicting engagement**			
**FLCA → Engagement (total effect)**	**–0.487**	**0.048**	**[ –0.581, –0.393 ]**
**Model B: parallel mediation with cognitive reappraisal and**			
**expressive suppression**			
Direct: FLCA → Cognitive reappraisal	–0.324	0.055	[ –0.432, –0.216 ]
Direct: FLCA → Expressive suppression	0.300	0.058	[ 0.186, 0.414 ]
Direct: Cognitive reappraisal → Engagement	0.379	0.060	[ 0.261, 0.497 ]
Direct: Expressive suppression → Engagement	–0.250	0.057	[ –0.362, –0.138 ]
Direct: FLCA → Engagement	–0.289	0.062	[ –0.411, –0.167 ]
Indirect: FLCA → Engagement via cognitive reappraisal	–0.123	0.032	[ –0.185, –0.060 ]
Indirect: FLCA → Engagement via expressive suppression	–0.075	0.029	[ –0.132, –0.018 ]
Total indirect effect	–0.198	0.040	[ –0.276, –0.119 ]
Total: FLCA → Engagement	–0.487	0.050	[ –0.581, –0.393 ]

Standardized effects are based on structural equation models estimated with robust maximum likelihood. Model A includes only the direct path from FLCA to English learning engagement (total effect). Model B includes both cognitive reappraisal and expressive suppression as parallel mediators and estimates direct, specific indirect, and total indirect effects. Indirect and total effects are based on bias-corrected bootstrap confidence intervals (5,000 resamples) All effects are standardized estimates from latent-variable SEMs in which each construct was specified as a latent factor indicated by its items.

FLCA, foreign language classroom anxiety.

## 3 Results

### 3.1 Descriptive statistics and correlations among key variables

Table 2 presents the means, standard deviations, and correlations among the four focal constructs. On average, students reported moderate levels of FLCA (*M* = 3.05, *SD* = 0.63), moderate use of cognitive reappraisal in English learning (*M* = 3.45, *SD* = 0.58) and expressive suppression (*M* = 3.11, *SD* = 0.64), and moderately high levels of English learning engagement (*M* = 3.60, *SD* = 0.61). The correlation pattern was broadly consistent with the hypothesized relationships. FLCA showed a moderate negative correlation with learning engagement (*r* = −0.49) and a smaller negative correlation with cognitive reappraisal (*r* = −0.32), while being positively correlated with expressive suppression (*r* = 0.30). Cognitive reappraisal was positively associated with engagement (*r* = 0.50), whereas expressive suppression was negatively associated with engagement (*r* = −0.37) and weakly negatively correlated with cognitive reappraisal (*r* = −0.10). Overall, these small-to-moderate correlations support examining the proposed parallel mediation model in the subsequent structural equation analyses.

### 3.2 Measurement model

To examine the measurement properties of the four latent constructs, we first estimated a four-factor confirmatory factor analysis with FLCA, cognitive reappraisal in English learning, expressive suppression in English learning, and English learning engagement specified as correlated latent variables. The measurement model demonstrated a good fit to the observed data: $\chi_{\text{SB}}^{2}=284.56$, *df* = 146, *p* < 0.001, *CFI* = 0.952, *TLI* = 0.944, *RMSEA* = 0.049 [90% CI: 0.041, 0.058], and *SRMR* = 0.042. As shown in Table 3, all items loaded significantly on their intended factors, with standardized loadings generally falling in the moderate-to-high range (approximately 0.69–0.83). For FLCA and engagement, factor loadings ranged from about 0.68 to 0.83, and for cognitive reappraisal and expressive suppression, loadings were between about 0.70 and 0.80. All factor loadings were statistically significant at *p* < 0.001, indicating that each item made a meaningful contribution to its corresponding latent construct.

Internal consistency and convergent validity evidence was also satisfactory. Composite reliability values ranged from 0.88 to 0.93, and AVE values ranged from 0.54 to 0.61. Composite reliability (CR) indicated adequate internal consistency, and AVE supported convergent validity (AVE >0.50) (Fornell and Larcker, 1981). Overall, the measurement model supported the intended four-factor structure and justified the use of these latent variables in the subsequent structural equation modeling analyses.

### 3.3 Structural model and mediation

We then estimated a baseline total-effect model (Model A) in which engagement was regressed on FLCA only, without specifying the mediation regressions involving the two emotion regulation factors. Model B extended this baseline model by adding the parallel mediation paths from FLCA to the two strategies and from the two strategies to engagement. Model A yielded the following fit indices: $\chi_{\text{SB}}^{2}=356.78$, *df* = 152, *p* < 0.001, *CFI* = 0.931, *TLI* = 0.925, *RMSEA* = 0.061 [90% CI: 0.053, 0.069], and *SRMR* = 0.062. Model A and Model B were estimated as nested latent-variable SEMs that retained the same four-factor measurement structure as the CFA; the models differed only in their structural regressions. Specifically, Model A estimated the FLCA → engagement association without specifying the mediation regressions, whereas Model B added these mediation paths to test the parallel mediation hypotheses. As shown in Table 4, FLCA had a moderate and statistically significant negative total effect on engagement (β = −0.487, *SE* = 0.048, 95% CI [–0.581, –0.393]). This result indicates that, taken as a whole, higher levels of foreign language classroom anxiety are associated with substantially lower levels of students' energy, dedication, and absorption in English learning, thereby supporting H1. Building on this baseline model, Model B introduced cognitive reappraisal and expressive suppression in English learning as parallel mediators (Figure 2). The mediation model (Model B) demonstrated a robust fit and, descriptively, a better fit than Model A: $\chi_{\text{SB}}^{2}=312.18$, *df* = 148, *p* < 0.001, *CFI* = 0.946, *TLI* = 0.938, *RMSEA* = 0.053 [90% CI: 0.045, 0.062], and *SRMR* = 0.048. In this model, FLCA significantly predicted both emotion regulation strategies. Consistent with H2a, higher FLCA was associated with less frequent use of cognitive reappraisal (β = −0.324, *SE* = 0.055, 95% CI [–0.432, –0.216]). In line with H2b, FLCA was positively related to expressive suppression (β = 0.300, *SE* = 0.058, 95% CI [0.186, 0.414]). These paths suggest that more anxious students tend to engage less in reframing difficult English learning situations in a constructive way and more in hiding or inhibiting their emotional reactions.

In turn, both emotion regulation strategies were significantly linked to English learning engagement. As predicted by H3a, cognitive reappraisal showed a positive association with engagement (β = 0.379, *SE* = 0.060, 95% CI [0.261, 0.497]), indicating that students who more often reinterpret emotionally challenging situations in English learning tend to feel more vigorous, dedicated, and absorbed. Conversely, in line with H3b, expressive suppression was negatively associated with engagement (β = −0.250, *SE* = 0.057, 95% CI [–0.362, –0.138]), suggesting that frequently hiding one's anxiety or discomfort in English classes is linked to lower engagement. Even after including both mediators, FLCA retained a significant negative direct effect on engagement (β = −0.289, *SE* = 0.062, 95% CI [–0.411, –0.167]). Thus, higher FLCA is related to lower engagement not only through its impact on emotion regulation strategies but also via a remaining direct pathway, indicating partial rather than full mediation. Regarding the indirect effects, both mediation pathways were statistically significant. The indirect effect of FLCA on engagement via cognitive reappraisal (H4a) was β = −0.123 (*SE* = 0.032, 95% CI [–0.185, –0.060]), and the indirect effect via expressive suppression (H4b) was β = −0.075 (*SE* = 0.029, 95% CI [–0.132, –0.018]). The total indirect effect was β = −0.198 (*SE* = 0.040, 95% CI [–0.276, –0.119]), accounting for roughly two fifths of the overall association between FLCA and engagement. Within this total indirect effect, the pathway through cognitive reappraisal was somewhat stronger than the pathway through expressive suppression, suggesting that difficulties in engaging in adaptive reappraisal may play a particularly important role in how anxiety undermines engagement.

The pattern of direct and indirect effects in Model B (Table 4; Figure 2) provides converging support for all four sets of hypotheses. FLCA is linked to lower English learning engagement both directly and through its associations with less frequent cognitive reappraisal and more frequent expressive suppression in English learning, with the two emotion regulation strategies functioning as complementary, partially mediating mechanisms.

### 3.4 Robustness checks: covariate-adjusted model

To evaluate whether the proposed mediation pattern could be attributable to background differences, we estimated an additional covariate-adjusted model in which gender, years of learning English, and self-rated English proficiency predicted the two mediators and English learning engagement. As shown in Table 5, the focal pattern of effects was substantively unchanged: FLCA remained negatively associated with cognitive reappraisal (β = −0.312, 95% CI [–0.416, –0.208]) and positively associated with expressive suppression (β = 0.288, 95% CI [0.178, 0.398]); cognitive reappraisal was positively, and expressive suppression negatively, related to engagement (β = 0.365, 95% CI [0.249, 0.481]; β = −0.238, 95% CI [–0.346, –0.130]). Both specific indirect effects remained significant (via cognitive reappraisal: β = −0.114, 95% CI [–0.173, –0.055]; via expressive suppression: β = −0.069, 95% CI [–0.122, –0.016]), supporting the robustness of the parallel mediation account. Among the covariates, self-rated proficiency and years of learning English showed small positive associations with engagement, whereas the gender effect was not statistically significant (Table 5).

**Table 5:** Robustness check: mediation model adjusted for covariates.

**Path/effect**	**Standardized**	***SE***	**95% CI**
**Model C: focal structural paths (adjusted for covariates)**			
Direct: FLCA → Cognitive reappraisal	–0.312	0.053	[ –0.416, –0.208 ]
Direct: FLCA → Expressive suppression	0.288	0.056	[ 0.178, 0.398 ]
Direct: Cognitive reappraisal → Engagement	0.365	0.059	[ 0.249, 0.481 ]
Direct: Expressive suppression → Engagement	–0.238	0.055	[ –0.346, –0.130 ]
Direct: FLCA → Engagement	–0.275	0.060	[ –0.393, –0.157 ]
Indirect: FLCA → Engagement via cognitive reappraisal	–0.114	0.030	[ –0.173, –0.055 ]
Indirect: FLCA → Engagement via expressive suppression	–0.069	0.027	[ –0.122, –0.016 ]
Total indirect effect	–0.183	0.038	[ –0.257, –0.109 ]
Total: FLCA → Engagement	–0.458	0.049	[ –0.554, –0.362 ]
**Control variables (effects on cognitive reappraisal)**			
Gender (Female = 1) → Cognitive reappraisal	0.028	0.046	[ –0.062, 0.118 ]
Years of learning English → Cognitive reappraisal	0.065	0.044	[ –0.021, 0.151 ]
Self–rated proficiency → Cognitive reappraisal	0.142	0.050	[ 0.044, 0.240 ]
**Control variables (effects on expressive suppression)**			
Gender (Female = 1) → Expressive suppression	–0.125	0.048	[ –0.219, –0.031 ]
Years of learning English → Expressive suppression	–0.034	0.045	[ –0.122, 0.054 ]
Self–rated proficiency → Expressive suppression	–0.088	0.052	[ –0.190, 0.014 ]
**Control variables (effects on engagement)**			
Gender (Female = 1) → Engagement	0.042	0.045	[ –0.046, 0.130 ]
Years of learning English → Engagement	0.085	0.042	[ 0.003, 0.167 ]
Self-rated proficiency → Engagement	0.194	0.051	[ 0.094, 0.294 ]

Model estimates the parallel mediation model while controlling for gender, years of formal English learning, and self-rated English proficiency. These covariates were specified to predict the mediators and engagement.

## 4 Discussion

### 4.1 Summary and theoretical interpretation of key findings

The present study examined how foreign language classroom anxiety (FLCA) is linked to English learning engagement among Chinese university students and whether two emotion regulation strategies, cognitive reappraisal and expressive suppression in English learning, help explain this association. In line with classic work conceptualizing FLCA as a situation-specific form of debilitating anxiety that interferes with language processing and performance (Horwitz et al., 1986), students in our sample reported moderate levels of FLCA alongside moderately high levels of English learning engagement. The negative zero-order association between FLCA and engagement is consistent with meta-analytic and review evidence indicating that higher FLCA is reliably associated with lower achievement, less favorable self-perceptions, and weaker engagement with the L2 (Botes et al., 2020; Shen, 2021; Zeng, 2021). Similar patterns have also been reported in recent L2 research showing that higher anxiety is associated with reduced willingness to communicate, lower participation, and less sustained classroom involvement, behavioral tendencies that are conceptually close to the energetic and participatory facets of engagement (Tsang and Dewaele, 2024; Zhao, 2023).

Our structural models provided a more fine-grained picture of these relationships. In the baseline model, FLCA exerted a substantial negative total effect on English learning engagement, echoing previous findings that anxiety is one of the strongest affective predictors of lower L2 grades, self-perceived competence, and willingness to communicate (Botes et al., 2020; Tsang and Dewaele, 2024; Zhao, 2023; Li, 2024). Beyond the well-established anxiety–achievement narrative, this result highlights English learning engagement as a consequential motivational state that FLCA may systematically undermine, suggesting that anxiety can be detrimental not only for immediate performance but also for learners' sustained investment of effort and attention in English learning (Botes et al., 2020; Shen, 2021; Zeng, 2021).

The parallel mediation analysis further clarified the internal mechanisms underlying this association and constitutes the central theoretical contribution of the present study. Specifically, students with higher FLCA reported using cognitive reappraisal less frequently and expressive suppression more frequently; in turn, cognitive reappraisal was positively associated with engagement, whereas expressive suppression was negatively associated with engagement. Interpreted through the emotion-regulation process model (Gross and John, 2003), these findings support a process-oriented dual-route account: reappraisal, as an antecedent-focused strategy, may help learners reinterpret demanding English-learning situations (e.g., mistakes, negative feedback, or high-stakes tasks) early in the emotion-generative process, thereby reducing the intensity of negative affect and preserving motivational and attentional resources that support vigor, dedication, and absorption. By changing the meaning of the situation, reappraisal may maintain perceived controllability and task value and free working-memory resources for task-focused processing (Gross and John, 2003). Consistent with this account, engagement-focused evidence in educational contexts indicates that adaptive cognitive emotion-regulation strategies such as reappraisal are positively linked to students' academic engagement (Buzdar and Ikram, 2024; Alkharj et al., 2024). In contrast, suppression, as a response-focused strategy, involves inhibiting outward emotional expression after the emotion has been elicited and can require ongoing self-monitoring. This inhibition may impose cognitive load, disrupt concentration and immersion, and reduce authentic participation in classroom activities—processes that are central to behavioral and emotional facets of engagement (Gross and John, 2003; Wong and Liem, 2022). Accordingly, engagement-relevant evidence suggests that greater reliance on suppression tends to coincide with poorer engagement-related functioning, whereas reappraisal shows the opposite pattern (Alkharj et al., 2024). This dual-pathway pattern is also consistent with meta-analytic evidence that reappraisal is generally associated with better adjustment, whereas suppression shows a less adaptive profile (Aldao et al., 2010; Webb et al., 2012), and it resonates with higher-education studies linking reappraisal to engagement and resilience and linking suppression-related difficulties to exhaustion and disengagement (Buzdar and Ikram, 2024; Iuga et al., 2025; Huang et al., 2023). Taken together, these results position emotion regulation as a proximal bridge between situation-specific FLCA and sustained engagement, while also leaving room for additional mechanisms that may account for the remaining direct association.

Finally, the present findings can be situated within broader theories of academic emotions. Control–value theory proposes that anxiety is likely when learners perceive high task importance but low control, whereas engagement is fostered by more facilitating appraisals and *positive activating emotions* (e.g., enjoyment, hope) (Pekrun, 2006). The broaden-and-build theory similarly contends that positive emotions broaden thought–action repertoires and build enduring resources over time (Fredrickson, 2001). In this light, cognitive reappraisal in English learning may help anxious students shift appraisals in a more adaptive direction and thereby sustain vigor, dedication, and absorption, whereas expressive suppression may constrain behavioral and cognitive involvement by limiting outward participation and narrowing engagement-related actions. This interpretation aligns with positive-psychology work in SLA showing that enjoyment and other positive emotions support learners' willingness to take risks, experiment with the language, and invest effort in L2 activities (Wu and Kabilan, 2025; Dewaele and MacIntyre, 2014; Li et al., 2018; Zeng, 2021; Resnik and Dewaele, 2020; Mohammad Hosseini et al., 2022; Derakhshan and Fathi, 2024). It also converges with research on academic and work engagement more broadly, where vigor, dedication, and absorption are sustained by supportive environments and adaptive emotional experiences (Schaufeli et al., 2002; Carmona-Halty et al., 2019; Martin and Bolliger, 2018). In this sense, our study highlights emotion regulation as a theoretically meaningful mechanism linking classroom-specific anxiety to the broader motivational state of engagement in L2 learning.

### 4.2 Practical implications

The findings also carry several implications for educational practice in university English-as-a-foreign-language settings. First, the strong negative association between FLCA and engagement underscores the importance of classroom environments that minimize unnecessary anxiety and enhance students' sense of control and competence. Prior intervention and descriptive work suggests that structured opportunities for low-stakes speaking, teacher immediacy, supportive feedback, and explicit discussion of language anxiety can reduce FLCA and foster more positive attitudes toward L2 learning (Horwitz et al., 1986; MacIntyre and Gregersen, 2012; Dewaele and MacIntyre, 2014; Li et al., 2018; Zeng, 2021). Our results suggest that such initiatives may be particularly beneficial when they also support students in developing adaptive ways of interpreting and responding to emotionally challenging situations in English classes.

Second, the mediating role of cognitive reappraisal points to the value of explicitly teaching and practicing this strategy in L2 classrooms. Instructors can guide students to view mistakes as informative feedback, reframe demanding tasks as manageable steps toward long-term goals, and reinterpret critical feedback as opportunities for growth rather than personal failure. Evidence from general education shows that brief reappraisal-focused interventions can enhance engagement, reduce test anxiety, and buffer the impact of stressors on academic outcomes (Cutuli, 2014; Buzdar and Ikram, 2024; Huang et al., 2023; Iuga et al., 2025). In English courses, such interventions might take the form of reflective journals in which students describe and reframe anxiety-provoking experiences, structured peer discussions about coping strategies, or teacher-led debriefings after particularly challenging communicative tasks. These activities could help students gradually internalize more constructive narratives about their emotions and capabilities in English learning.

Third, the negative association between expressive suppression and engagement suggests that encouraging students simply to “hide” their anxiety in class is unlikely to be productive. Instead, teachers might work toward normalizing emotional reactions in L2 learning, explicitly acknowledging that nervousness and embarrassment are common and understandable responses to speaking a foreign language. Research on emotional presence and social connectedness in higher education indicates that when instructors attend to and validate students' emotional experiences, students feel more supported and are more willing to participate actively (Shehzad and Charles, 2023; Martin and Bolliger, 2018; Miao and Ma, 2022). In practice, this could involve modeling disclosure of one's own language-learning struggles, inviting students to share their feelings about specific tasks, or establishing class routines in which students can express concerns or seek help without fear of ridicule. Such practices may reduce the perceived need for suppression and create a climate in which more adaptive forms of emotion regulation can flourish.

At a broader institutional level, the findings support the integration of wellbeing and emotion regulation components into university English programs. Workshops, counseling services, or co-curricular initiatives could be designed to help students recognize their emotional responses to language learning, practice cognitive reappraisal and related strategies, and explore alternatives to suppression such as problem-solving, seeking social support, or engaging in mastery-oriented goal setting. These efforts align with calls to place learner and teacher wellbeing at the center of language education (Mercer and Gregersen, 2020; Oxford, 2016; Dewaele and Li, 2021) and with evidence that emotionally supportive environments are crucial for sustaining engagement in both face-to-face and online learning (Martin and Bolliger, 2018; Dewaele and Li, 2021). In sum, our results suggest that attending to how students feel and how they regulate those feelings is not merely an adjunct to language teaching, but an integral part of fostering engaged, resilient English learners.

### 4.3 Limitations and future directions

Several limitations should be considered when interpreting the present findings. First, the cross-sectional design precludes strong inferences about temporal ordering and causality among FLCA, emotion regulation strategies, and engagement; longitudinal or diary/experience-sampling designs are needed to examine within-person dynamics over time. Second, because all focal constructs were measured via self-report questionnaires, future work could incorporate additional sources (e.g., teacher ratings) and behavioral indicators of engagement (e.g., participation and task completion) to reduce method-related concerns. Third, the single-university sample warrants caution regarding generalizability; replication across diverse institutions and learner populations would strengthen external validity. Finally, the current model focused on two regulation strategies, and future research could incorporate additional contextual and individual variables (e.g., classroom climate, teacher support, perceived competence) and test whether the mediating pathways vary across learner subgroups (e.g., gender or proficiency) using moderation or group-comparison analyses.

## 5 Conclusion

The present study investigated how foreign language classroom anxiety (FLCA) is associated with English learning engagement among Chinese university students and examined the mediating roles of two emotion regulation strategies in English learning: cognitive reappraisal and expressive suppression. Using structural equation modeling with latent variables, we found that FLCA was substantially and negatively related to English learning engagement. Students who felt more anxious in their English classes reported lower levels of vigor, dedication, and absorption in learning. At the same time, higher FLCA was linked to less frequent use of cognitive reappraisal and more frequent use of expressive suppression, and these strategies were in turn positively and negatively associated with engagement, respectively.

The mediation analyses showed that cognitive reappraisal and expressive suppression jointly and partially explained the negative association between FLCA and engagement. Anxious students were less likely to reinterpret emotionally challenging English learning situations in a constructive way and more likely to hide or inhibit their emotional reactions, and both tendencies were associated with reduced engagement. The indirect pathway through cognitive reappraisal was somewhat stronger than the pathway through expressive suppression, suggesting that fostering adaptive reappraisal may be particularly important for sustaining engagement in the face of anxiety. Nevertheless, a sizeable direct effect of FLCA on engagement remained, indicating that emotion regulation is one key mechanism among others linking anxiety to engagement.

These findings highlight the importance of addressing both emotional experiences and emotion regulation processes in university English-as-a-foreign-language settings. Efforts to reduce excessive FLCA, to strengthen students' capacity to reappraise stressful learning situations, and to provide alternatives to expressive suppression may help learners remain more energetically and meaningfully engaged with English. By foregrounding emotion regulation as a bridge between classroom anxiety and engagement, the study contributes to a more comprehensive understanding of how students feel, cope, and ultimately participate in foreign language learning at the tertiary level. Future research could extend the present framework by explicitly examining whether the proposed pathways differ across learner characteristics (e.g., gender or proficiency-related indicators) and by testing potential moderation processes in designs that allow for stronger inferences about conditional effects.

## Data Availability

The original contributions presented in the study are included in the article/Supplementary material, further inquiries can be directed to the corresponding author.
