# Supplementary material for: From foreign language classroom anxiety to English learning engagement: the roles of cognitive reappraisal and expressive suppression
Source: Front Psychol. 2026 Mar 10;17:1754113. doi: 10.3389/fpsyg.2026.1754113 (PMC13008866; doi:10.3389/fpsyg.2026.1754113)
Supplement: Supplementary file 1 [file Supplementary_file_1.pdf]

Supplementary Material: Appendix 1

Full Item List

Response format. All items were rated on a 5-point Likert scale (1 = strongly disagree, 5 = strongly agree).
Administration note. All items were administered in Chinese; English renderings below are provided for reporting purposes.

A1. Foreign Language Classroom Anxiety (FLCA; 8 items)

Code	Chinese item (administered)	English rendering
FLCA1	当老师在英语课上点名让我回答时，我会感到紧张。	When the teacher calls on me in English class, I feel nervous.
FLCA2	在英语课上开口说英语时，我担心自己会说错。	When speaking English in class, I worry that I will make mistakes.
FLCA3	我担心同学会因为我英语说得不好而笑我。	I worry that classmates might laugh at me if my English is not good.
FLCA4	需要即兴用英语表达时，我会感到不安。	I feel uneasy when I have to express myself in English without preparation.
FLCA5	英语课堂测验或考试时，我会明显焦虑。	I feel noticeably anxious during English quizzes or tests.
FLCA6	我担心老师会对我的英语表现做出负面评价。	I worry that the teacher will evaluate my English performance negatively.
FLCA7	即使我提前准备了英语课内容，上课时仍会感到紧绷。	Even when I have prepared, I still feel tense in English class.
FLCA8	看到别人英语更流利时，我会感到压力和不自在。	When others seem more fluent, I feel pressure and discomfort.

A2. Cognitive Reappraisal in English Learning (CR; 6 items)

Code	Chinese item (administered)	English rendering
CR1	当英语学习让我心情不好时，我会尝试换个角度看待。	When English learning makes me feel bad, I try to look at it from a different perspective.
CR2	英语学习让我有压力时，我会提醒自己这是正常的、会过去的。	When I feel stressed about English learning, I remind myself it is normal and temporary.
CR3	在英语学习中犯错时，我会把它看作改进的机会。	When I make mistakes in English learning, I view them as opportunities to improve.
CR4	课堂上感到焦虑时，我会把注意力放在自己能控制的事情上，让自己更平静。	When I feel anxious in class, I focus on what I can control to calm myself down.
CR5	收到英语学习方面的负面反馈时，我会把它理解为有帮助的建议。	When I receive negative feedback about my English, I interpret it as helpful guidance.
CR6	当英语任务很难时，我会尝试用更积极的方式重新理解这种困难。	When English tasks feel difficult, I try to reframe the difficulty in a more positive way.

A3. Instructed-Response Attention Check (1 item)

Code	Chinese item (administered)	English rendering
AC1	为了确认你在认真作答，请在本题选择“5（非常同意）”。	To show that you are paying attention, please select “5 (strongly agree)” for this item.

A4. Expressive Suppression in English Learning (ES; 6 items)

Code	Chinese item (administered)	English rendering
ES1	在英语课上感到紧张时，我会尽量不让别人看出来。	When I feel nervous in English class, I try not to show it to others.
ES2	听不懂英语内容而感到沮丧时，我会把这种情绪藏起来。	When I feel frustrated because I cannot understand, I hide that emotion.
ES3	即使我在课堂上感到尴尬，我也会保持表情镇定。	Even if I feel embarrassed in class, I keep my expression calm.
ES4	英语课堂活动让我焦虑时，我会刻意控制自己的情绪外露。	When class activities make me anxious, I deliberately control outward emotional expression.
ES5	进行英语展示或发言时，我会压住紧张情绪，不表现出来。	During English presentations or speaking, I suppress nervousness and do not show it.
ES6	在英语学习过程中，我倾向于把情绪留给自己，不让别人知道。	In English-learning situations, I tend to keep my feelings to myself.

A5. English Learning Engagement (ENG; 9 items)

Code	Chinese item (administered)	English rendering
ENG1	学习英语时，我感到精力充沛。	When I study English, I feel full of energy.
ENG2	即使英语学习很有挑战，我也能持续投入。	Even when English learning is demanding, I can keep going.
ENG3	我愿意在英语学习上投入额外的努力。	I am willing to invest extra effort in English learning.
ENG4	我觉得英语学习对我很有意义。	I find English learning meaningful.
ENG5	我对提高英语水平感到热情和期待。	I feel enthusiastic about improving my English.
ENG6	当我在英语学习上取得进步时，我会感到自豪。	I feel proud when I make progress in English learning.
ENG7	学习英语时，我常常全身心投入其中。	When learning English, I often become fully immersed.
ENG8	我学习英语时，时间常常过得很快。	Time passes quickly when I study English.
ENG9	一旦开始做英语学习任务，我常常很难马上停下来。	Once I start an English-learning task, it is often hard for me to stop right away.
